# Interplay of Fracture and Martensite Transformation in Microstructures: A Coupled Problem

**DOI:** 10.3390/ma15196744

**Published:** 2022-09-28

**Authors:** Ehsan Borzabadi Farahani, Behnam Sobhani Aragh, Daniel Juhre

**Affiliations:** 1 Department of Wind Energy, Technical University of Denmark, Frederiksborgvej 399, 4000 Roskilde, Denmark; 2 Institute of Mechanics, Faculty of Mechanical Engineering, Otto von Guericke University Magdeburg, Universitätsplatz 2, 39106 Magdeburg, Germany; 3 School of Computing, Engineering and Digital Technologies, Teesside University, Tees Valley, Middlesbrough TS1 3BX, UK

**Keywords:** phase-field approach, coupled problem, fracture mechanics, finite element method, crack growth, martensitic phase transformation

## Abstract

We are witnessing a tremendous transition towards a society powered by net-zero carbon emission energy, with a corresponding escalating reliance on functional materials (FM). In recent years, the application of FM in multiphysics environments has brought new challenges to the mechanics and materials research communities. The underlying mechanism in FM, which governs several fundamental characteristics, is known as martensitic phase transformation (MPT). When it comes to the application of FM in the multiphysics context, a thorough understanding of the interplay between MPT and fracture plays a crucial role in FM design and application. In the present work, a coupled problem of crack nucleation and propagation and multivariant stress-induced MPT in elastic materials is presented using a finite element method based on Khachaturyan’s microelasticity theory. The problem is established based on a phase-field (PF) approach, which includes the Ginzburg–Landau equations with advanced thermodynamic potential and the variational formulation of Griffith’s theory. Therefore, the model consists of a coupled system of the Ginzburg–Landau equations and the static elasticity equation, and it characterizes evolution of distributions of austenite and two martensitic variants as well as crack growth in terms of corresponding order parameters. The numerical results show that crack growth does not begin until MPT has grown almost completely through the microstructure. Subsequent to the initial formation of the martensite variants, the initial crack propagates in such a way that its path mainly depends on the feature of martensite variant formations, the orientation and direction upon which the martensite plates are aligned, and the stress concentration between martensite plates. In addition, crack propagation behavior and martensite variant evaluations for different lattice orientation angles are presented and discussed in-detail.

## 1. Introduction

The transition towards a net-zero carbon emissions system, electro-mobility, and sustainable energy comes with an escalating demand for functional materials (FM) with wide application in electric motors, generators, robotics, automation, and storage devices [1]. In particular, these materials play a crucial role in the development of environmentally friendly solid-state refrigerators, addressing increasing energy demand for cooling [2]. The underlying mechanism in FM that governs several fundamental phenomena, such as multicaloric effects, shape-memory effects, pseudoelasticity, and pseudoplasticity, is known as the martensitic phase transformation (MPT) [3,4]. MPT is a first-order transformation during which no diffusion occurs and *i*th martensitic variants can transform to each other owing to thermal or mechanical loadings and alteration in the surface energy. For the development of FM, two points must be highlighted: On the one hand, the high rate of MPT and the complexities associated with conducting in situ analysis of the process make it cumbersome to investigate MPT under different loading conditions and, in particular, to design and optimize the material system. On the other hand, the application of FM in multiphysics environment has triggered new research areas. In particular, crucial attention should be paid to the interaction between MPT and fractures, which is of great importance in material science and engineering. In the last years, the nucleation and growth of cracks in brittle austenitic microstructures has been investigated in the literature using several experimental techniques, mainly at the macroscale. Nevertheless, a thorough understanding of the fundamentals of crack nucleation and growth in austenitic microstructures at the microscale is highly imperative.

In recent years, the phase-field (PF) approach has proved to be a powerful computational method for modeling and tracking microstructural and morphological evolution in materials at the mesoscale [5,6,7]. Materials scientists commonly aim to enhance material properties by an in-depth understanding of the predominant mechanisms driving microstructural transformations. These transformations are fundamentally dependent on the composition and topology of each microstructural feature [8]. Therefore, a comprehensive understanding of the mechanics of these transformations can provide accurate predictions and enhanced reliability of microstructural design. Various PF models have been developed and employed for modeling microstructural evolutions, such as solidification [9,10], precipitate growth and coarsening [11], oxidation [12,13], grain growth [14,15], and MPT. Based on a PF model, first, a set of conserved and/or non-conserved field variables are introduced, which smoothly vary across the interfacial regions separating adjacent phases. Second, the evolution of these field variables is described by employing the temporal and spatial evolution of the PF variables, which are governed by the Cahn–Hilliard nonlinear diffusion equation [16] and the time-dependent Ginzburg–Landau (TDGL) relaxation equation [17,18].

An infinitely sharp interface between the austenite and martensite phases in the MPT can be addressed by regularizing the discontinuities by means of a PF model, in contrast to the technique of tracking moving interfaces used by Cherkaoui et al. [19]. Levitas and Preston [20,21] presented a Landau theory for multivariant stress-induced MPT. Based on microelasticity theory, a multi-scale PF approach to MPT was introduced by Wang and Khachaturyan [22,23]. Furthermore, a PF model to study the structural mechanism of heterogeneous initiation and propagation in the face-centered cubic (fcc) to body-centered cubic (bcc) martensitic transformation was presented by Zhang et al. [24]. An elasto–plastic PF model was developed to model the evolution of the martensitic microstructure in a single crystal [25] and a polycrystal [26]. The model was based on the PF microelasticity theory [27] coupled with the plasticity model developed by Guo et al. [28]. Schmitt et al. [29] presented a PF model for multi-variant martensitic transformations of stable and metastable phases. It was concluded that the martensitic phases form in compliance with theoretical studies and crystallographic theories, while the nucleation characteristics of the model is adaptable. Mamivand et al. [30] developed a 2D PF model to predict microstructural evolution during the tetragonal-to-monoclinic phase transformation in zirconia. Inhomogeneous and anisotropic elastic properties were considered in the model, and governing equations were solved in a finite element (FE) framework. A PF model was developed by Xie et al. [31] to simulate the cyclic phase transition of the single-crystal NiTi shape memory alloy with super-elasticity. Babaei et al. [32] introduced a PF approach for stress-induced MPT that considers the crystal lattice instability conditions obtained by atomistic simulations. The shape memory effect and pseudoelasticity of polycrystalline shape memory alloys with consideration of the latent heat were investigated by Sun et al. [33]. In that work, the latent heat release and absorption accompanying the phase transformation processes were explicitly considered by coupling the PF evolution with latent heat conduction. An elasto–plastic PF model to study the mechanics of tetragonal-to-monoclinic phase transformation and elasto–plastic deformation of polycrystalline yttria-stabilized tetragonal zirconia was developed by Cissé and Asle Zaeem [34]. The impact of a pre-existing nanovoid on multi-variant martensitic transformation was investigated by Javanbakht and Ghaedi [35]. In order to create a pre-existing nanovoid in the model, a single nanovoid was stabilized in the center of the computational domain using a PF approach. Most recently, a PF approach was presented by Borzabadi Farahani et al. [36] to study crack nucleation and propagation in martensitic microstructures resulting from multi-variant MPT within the framework of an FEM.

Comparatively large transformation strain induces a large stress concentration, which may be relaxed by crack and void nucleation and propagation rather than plasticity. At the same time, a high stress concentration at the crack tip may cause MPT [37,38,39]. Both increase the resistance to crack growth and ductility, which is called transformation toughening. The PF approach for crack initiation and growth [40], in which crack paths are automatically determined as part of the solution, has been developed in the physics community [41,42,43,44] and the mechanics community [45,46,47,48,49,50,51]. The former employed the Ginzburg–Landau [52] formalism to model crack growth; however, the latter used the variational formulation of classical Griffith’s theory of brittle fracture primarily established by Francfort and Marigo [53]. Interactions between MPT and fracture are a remarkably crucial problem in mechanics of strength and deformational and transformational properties of materials. Despite the utmost importance of the problem, only a few studies considered both MPT and fracture as a coupled system with the PF approach. A PF theory incorporating both fracture and deformation twinning behaviors in crystalline solids was described and implemented in finite element calculations by Clayton and Knap [54]. Their result showed a tendency for fracture before twinning when surface energies of the two mechanisms are equal, and a tendency for twining to delay fracture when the fracture energy substantially exceeds the twined boundary energy. A combined continuum PF model for MPT and fracture was introduced by Schmitt et al. [55]. In their work, only one-variant MPT was studied. The effect of MPT combined with crack initiation and propagation was compared with crack growth behavior in a purely austenitic specimen. They reported that because of the volume change and lattice distortion during the MPT, an eigenstrain in the martensitic phase increases, which leads to a different stress field compared to that of a homogeneous austenitic specimen with the same load applied. A PF approach for the interaction of fracture and MPT was developed by Jafarzadeh et al. [56] that includes the change in surface energy during MPT and the impact of unexplored scale parameters proportional to the ratio of the widths of the crack surface and the phase interface, both at the nanometer scale. Zhao et al. [57] investigated the tetragonal-to-monoclinic phase transformation and its toughening effect on Mode I crack propagation in single crystalline zirconia by a coupled PF model. The numerical results demonstrated that for both lattice orientations (0° and 90°), the phase transformation initiates at the crack tip. For $\theta =0^{0}$, the twining forms vertically, which is parallel to the crack, whereas for $\theta =90^{0}$, it grows horizontally. The evolution of twining in single-crystal magnesium was studied by Amirian et al. [58] using a PF model to gain better insight into the time-evolved twinned morphology, the spatial distribution of the internal shear stress, and the twinned interactions.

In this work, a coupled problem of crack nucleation and propagation and two-variant MPT is investigated based on PF approach-based FE formulations. The model established in the present study includes a coupled system of three TDGL questions that describe the evolution of the damage variable and two martensite variants in the quasi-static equilibrium equation. This work considers the positive dilatational component of transformation strain that accompanies the MPT from austenite to martensite phase and leads to an eigenstrain within the martensitic phase. Since the eigenstrain results in both tensile and compressive loads, the model takes the sign of the dilatational component into account. In particular, this study concentrates on the interactions between microcrack initiation and propagation and 2D phase transformation, which has not been reported so far according to our literature survey.

## 2. Fundamental Framework

In this work, two-variant martensitic microstructure includes austenite and two symmetry-related martensitic variants, which are represented in terms of the dispersion of two order parameters $c_{1}$ and $c_{2}$. Transformation strain $\varepsilon^{0}$ converts the crystal lattice of austenite into crystal lattice of *i*th martensitic variant. The temporal evolution of the order parameter $c_{i}$ can be presented by *i* TDGL equations, which express the linear relation between the rate of change of the order parameters and generalized thermodynamic forces conjugated to them. The TDGL equation for the order parameter $c_{i}$ is given by [22]

(1) $$\begin{array}{c} \frac{\partial_{c}}{\partial_{t}}=-M_{c}\frac{\delta \psi_{t}}{\delta c_{i}} \end{array}$$

where $M_{c}$ denotes a kinetic parameter, and δψtδψtδciδci is a variational derivative that determines the local driving force for martensite formation.

The total potential free energy of the system, $\psi_{t}$, is given as

(2) $$\begin{array}{c} \psi_{t}\left(\varepsilon ,c_{i},\nabla c_{i}\right)=W\left(\varepsilon ,c_{i}\right)+\psi^{grad}\left(\nabla c_{i}\right)+\psi^{sep}\left(c_{i}\right)+W^{es} \end{array}$$

where $W(\varepsilon ,c_{i})$ is the elastic strain energy density, $\psi^{grad}\left(\nabla c_{i}\right)$ denotes the gradient energy density, $W^{es}$ is the fracture energy density discussed later, and $\psi^{sep}\left(c_{i}\right)$ corresponds to the chemical free energy density of an unstressed system, which is expressed as

(3) $$\begin{array}{c} \psi^{sep}\left(c_{i}\right)=k_{sep}\frac{G_{c}}{L_{c}}f\left(c_{i}\right) \end{array}$$

where $G_{c}$ represents the characteristic interface energy density, $L_{c}$ dominates the width of the interface zone between the phases, and $k_{sep}$ represents a calibration constant. The function $f(c_{i})$ is a Landau polynomial expansion, where $f\left(c_{i}\right)=1+\frac{A}{2}{c_{i}}^{2}-\frac{B}{3}{c_{i}}^{3}+\frac{C}{4}{c_{i}}^{4}$. *A*, *B*, and *C* are the Landau polynomial expansion coefficients [36]. The gradient energy, defined as the sum of gradient energies due to the inhomogeneity of order parameters is expressed as [27,59]

(4) $$\begin{array}{c} \psi^{grad}\left(\nabla c_{i}\right)=\frac{1}{2}k_{grad}G_{c}L_{c}{∥\nabla c_{i}∥}^{2} \end{array}$$

where $k_{grad}$ is related to the interface energy between the phases and variants.

Contrary to chemical free energy, which assists the phase transformation, elastic strain energy must be overcome for MPT to progress. The source of the elastic strain energy during MPT is associated with the lattice mismatch between the different phases. Based on microelasticity theory, the strain energy is given by

(5) $$\begin{array}{c} W\left(\varepsilon ,c\right)=\frac{1}{2}\left[\varepsilon -\varepsilon^{0}\left(c_{i}\right)\right]:C\left(c_{i}\right)\left[\varepsilon -\varepsilon^{0}\left(c_{i}\right)\right] \end{array}$$

where $\varepsilon^{0}\left(c_{i}\right)=c_{1}\varepsilon_{1}^{0}+c_{2}\varepsilon_{2}^{0}$; $\varepsilon_{i}^{0}$ denotes the Bain strains. The material tensor $C(c_{i})$ denotes the elastic stiffness expressed by

(6) $$\begin{array}{c} C\left(c_{i}\right)=C_{A}+c_{1}\left(C_{M}-C_{A}\right)+c_{2}\left(C_{M}-C_{A}\right) \end{array}$$

where the indices *A* and *M* signify the austenitic and martensitic phase, respectively. Furthermore, ε is the linearized strain tensor related to the local displacement vector, u, given by

(7) $$\begin{array}{c} \varepsilon \left(u\right)=\frac{1}{2}\left(\nabla u+{\left(\nabla u\right)}^{T}\right) \end{array}$$

To inhibit crack interpenetration in compression, we break down the elastic strain into a positive volume change, ${\psi^{\bar{e}}}_{vol+}$, a negative volume change, ${\psi^{\bar{e}}}_{vol-}$, and a deviatoric part, ${\psi^{\bar{e}}}_{dev}$. This originated from the work of Amor et al. [47], in which the trace of the strain tensor was decomposed into positive and negative parts. As a result, the part of the elastic energy in regions with negative volume change cannot be released as a consequence of the creation of new crack surfaces. On the contrary, in the regions where volume change is positive, the elastic energy may contribute to the surface energy. Consequently, the elastic strain energy density is given by

(8) $$\begin{array}{c} W=W^{vol-}+W^{vol+}+W^{dev} \end{array}$$

where

(9) $$\begin{array}{c} W^{vol-}=\left\{\begin{array}{cc} \frac{K(c)}{2}tr{\left(\varepsilon -\varepsilon^{0}\left(c_{i}\right)\right)}^{2}, & \;tr(\varepsilon -\varepsilon^{0}\left(c_{i}\right))<0\\ 0, & \;\mathrm{else} \end{array}\right. \end{array}$$

(10) $$\begin{array}{c} W^{vol+}=\left\{\begin{array}{cc} \left(s^{2}+\zeta \right)\;\frac{K(c)}{2}tr{\left(\varepsilon -\varepsilon^{0}\left(c_{i}\right)\right)}^{2}, & \;tr(\varepsilon -\varepsilon^{0}\left(c_{i}\right))\geq 0\\ 0, & \;\mathrm{else} \end{array}\right. \end{array}$$

(11) $$\begin{array}{c} W^{dev}=\left(s^{2}+\zeta \right)\;\mu \left(c\right)\left[e-c_{i}e^{0}\right]:\left[e-c_{i}e^{0}\right] \end{array}$$

in which e=ε−tr(ε)tr(ε)22I and e0=ε0−tr(ε0)tr(ε0)22I are the deviatoric parts of the strain tensor and eigenstrain tensor in the 2D formulation, with I representing the 2D identity tensor. K(c) and μ(c) are the bulk and the shear modulus, respectively; ζ denotes residual stiffness to avoid instability in the numerical procedure.

**Figure 1:**
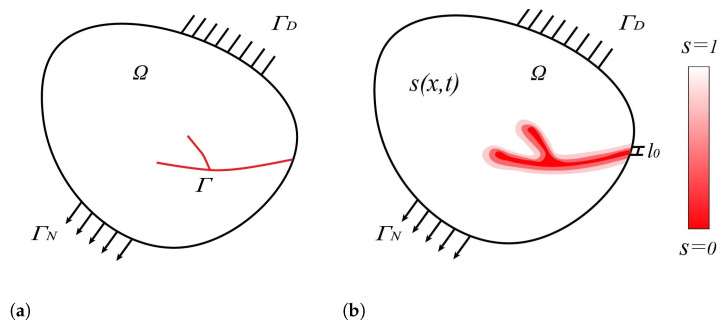
(**a**) Body with internal discontinuity (sharp crack), Γ; (**b**) approximation of internal discontinuity by a phase field model.

In this part, the PF model for fracture is presented based on the variational formulation of the Griffith’s theory introduced by Francfort and Marigo [53]. According to this model, the minimum energy required for creating a cracked surface per unit area is equivalent to the critical fracture energy density, termed the critical energy release rate. As depicted in Figure 1a, the body contains a crack, Γ, i.e., internal discontinuity. To approximate this jump in the PF approach, a damage parameter, *s*, is defined which is 1 in undamaged material and 0 at the crack. The so-called diffusive or regularized representation of the crack according to the PF approach is shown in Figure 1b. Following the work of Kuhn and Müller [46], the fracture energy density is expressed by

(12) $$\begin{array}{c} W^{es}=\frac{G_{s}{\left(1-s\right)}^{2}}{4L_{s}}+G_{s}L_{s}{∥\nabla s∥}^{2} \end{array}$$

in which $G_{s}$ denotes the critical strain energy release rate and $L_{s}$ is the length-scale parameter for the crack. Considering the contribution of the damage order parameter to the total PF potential, the total PF potential is decomposed in the following way

(13) $$\begin{array}{c} \psi_{t}=\underset{\psi_{ns}}{\underset{︸}{W^{vol-}+\psi^{grad}+\psi^{sep}}}+\underset{\psi_{s}}{\underset{︸}{W^{vol+}+W^{dev}+W^{es}}} \end{array}$$

where $\psi_{ns}$ is not coupled to the damage order parameter, *s*, in contrast to $\psi_{s}$. As a result, the elastic energy related to the negative volume change, $W^{vol-}$, cannot be minimized by generating cracks, which results in asymmetric outcomes in tension and compression situations. This dissimilarity is demanded since the Bain strain $\varepsilon^{0}\left(c_{i}\right)$ of the martensitic phase results in compression even if purely traction load is imposed.

Based on the TDGL equation for fracture, the evolution equation of the damage order parameter, *s*, can be written as

(14) $$\begin{array}{c} \frac{\partial_{s}}{\partial_{t}}=-M_{s}\frac{\delta \psi_{t}}{\delta s}=-M_{s}\left(2s\left(W^{vol+}+W^{dev}\right)-G_{s}\left(2L_{s}\Delta s+\frac{1-s}{2L_{s}}\right)\right) \end{array}$$

where $M_{s}$ denotes the mobility parameter scaling the kinetics of the crack growth.

On the other hand, the equilibrium equation of the body is given by

(15) $$\begin{array}{c} \nabla .\sigma =0 \end{array}$$

where the Cauchy stress tensor, σ, can be derived from the constitutive expression as

(16) $$\begin{array}{c} \sigma =\frac{\partial_{\psi_{t}}}{\partial_{\varepsilon }}=K\left(c\right)tr^{-}\left(\varepsilon -\varepsilon^{0}\left(c_{i}\right)\right)I+\left(s^{2}+\zeta \right)\left(K\left(c\right)tr^{+}\left(\varepsilon -\varepsilon^{0}\left(c_{i}\right)\right)I+2\mu \left(c\right)\left(e-e^{0}\left(c_{i}\right)\right)\right) \end{array}$$

## 3. Finite Element Implementation

In this section, the coupled model established in the previous section is implemented into a finite element framework with displacements u, MPT order parameter $c_{i}$, and damage order parameter *s*. With virtual displacements $\eta_{u}$ and virtual variables $\eta_{s}$ and $\eta_{c_{i}}$, the weak forms of Equations (1), (14) and (16) are, respectively,

(17) $$\begin{array}{c} \int_{\Omega }\nabla \eta_{u}:\sigma \;dV=\int_{\Gamma }\eta_{u}t^{*}dA \end{array}$$

(18) $$\begin{array}{c} \int_{\Omega }\frac{\dot{s}}{M_{s}}\eta_{s}\;dV-\int_{\Omega }q_{s}.\nabla \eta_{s}\;dV+\int_{\Omega }\left(2s\left(W^{vol+}+W^{dev}\right)-\frac{G_{s}\left(1-s\right)}{2L_{s}}\right)\eta_{s}\;d\Omega =-\int_{\Gamma }q_{s}^{*}\;\eta_{s}dA \end{array}$$

$$\int_{\Omega }\frac{{\dot{c}}_{i}}{M_{c}}\eta_{c_{i}}\;dV-\int_{\Omega }q_{c_{i}}.\nabla \eta_{c_{i}}\;dV+\int_{\Omega }\left(s^{2}+\zeta \right)\frac{\partial \left(W^{vol+}+W^{dev}\right)}{\partial c_{i}}\eta_{c_{i}}\;dV+\int_{\Omega }\frac{\partial \left(\psi^{sep}+W^{vol-}\right)}{\partial c_{i}}\eta_{c_{i}}\;dV$$

(19) $$=-\int_{\Gamma }q_{c}^{*}\;\eta_{c_{i}}dA$$

where $q_{c}=-k_{grad}G_{c}L_{c}\nabla c_{i}$ and $q_{s}=-G_{s}L_{s}\nabla s$. The discretization of $\eta_{u}$, $\eta_{s}$, and $\eta_{c_{i}}$ with shape functions $N_{I}$ for node *I* is expressed by

(20) $$\begin{array}{cc} u & =N_{I}u_{I}\\ c_{i} & =N_{I}c_{i}{}_{I}\\ \nabla c_{i} & =B_{I}^{c}{c_{i}}_{I}\\ \dot{s} & =N_{I}{\dot{s}}_{I} \end{array}\;\;\;\begin{array}{cc} \varepsilon & =B_{I}^{u}u_{I}\\ {\dot{c}}_{i} & =N_{I}{\dot{c}}_{i}{}_{I}\\ s & =N_{I}s_{I}\\ \nabla s & =B_{I}^{s}s_{I} \end{array}$$

In a 2D setting, the spatial derivatives can be expressed by means of the matrices

(21) $$\begin{array}{cc} B_{I}^{u} & =\left[\begin{array}{cc} N_{I,x} & 0\\ a & N_{I,y}\\ N_{I,y} & N_{I,x} \end{array}\right]\\ B_{I}^{s} & =\left[\begin{array}{c} N_{I,x}\\ N_{I,y} \end{array}\right] \end{array}\;\;\;\begin{array}{c} B_{I}^{c}=\left[\begin{array}{c} N_{I,x}\\ N_{I,y} \end{array}\right] \end{array}$$

Coupled Equations (1), (14), and (16) are implemented into the Finite Element Analysis Program (FEAP) within a finite element framework along with an implicit time integration scheme for the transient terms.

## 4. Numerical Results and Discussion

In this section, first, validation of the present formulation for one-variant MPT is presented. Afterwards, the PF model is applied to a coupled problem of two-variant MPT and fracture in a microstructure with an initial crack, and the results obtained are discussed in-detail.

**Figure 2:**
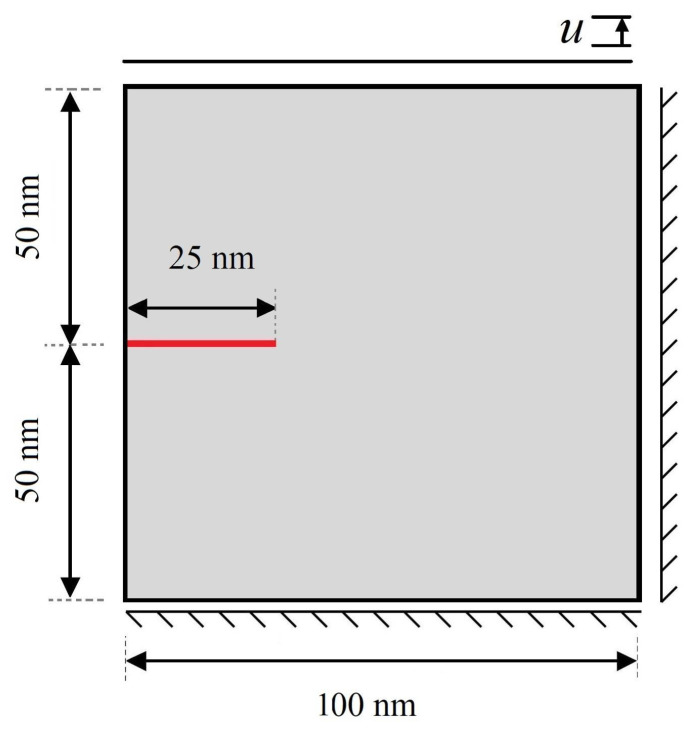
Initial configuration of an austenitic specimen with pre-existing crack under Mode I loading used for validation of the work.

**Figure 3:**
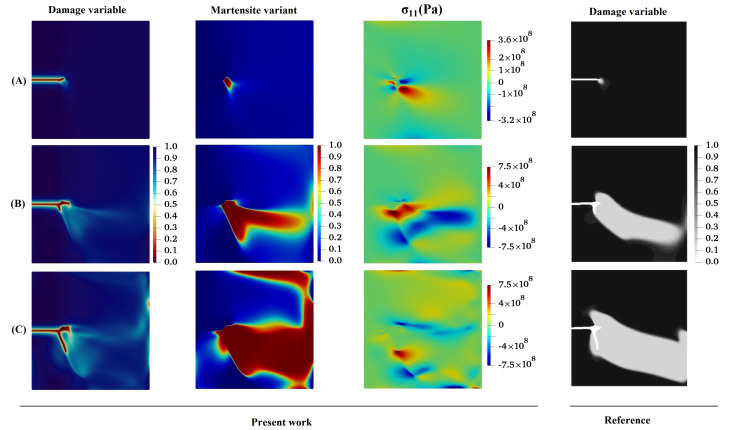
Contour plots of evolution of damage variable, martensite variant, and corresponding $\sigma_{11}$-component in a coupled problem of one-variant MPT and fracture.

Because of the lack of available experimental results for crack initiation and growth in microstructures subjected to one-variant MPT for direct comparison, the results obtained for an austenitic plate under Mode I loading are compared with those presented in [55]. A vertical displacement at the top and bottom surface in the *y*-direction is applied to the specimen to simulate a pure Mode I fracture, as shown in Figure 2. The evolutions of one-variant martensite phase and crack growth behavior obtained from the literature are compared with those from the present work, demonstrated in Figure 3. One can see that good agreement exists between the results of the present method and those obtained in [55]. As can be seen from Figure 3, the crack does not grow straight through the specimen but kinks and propagates in the vertical direction. Moreover, it is observed that crack growth does not begin until the one-variant martensite phase has propagated nearly entirely through the specimen.

**Figure 4:**
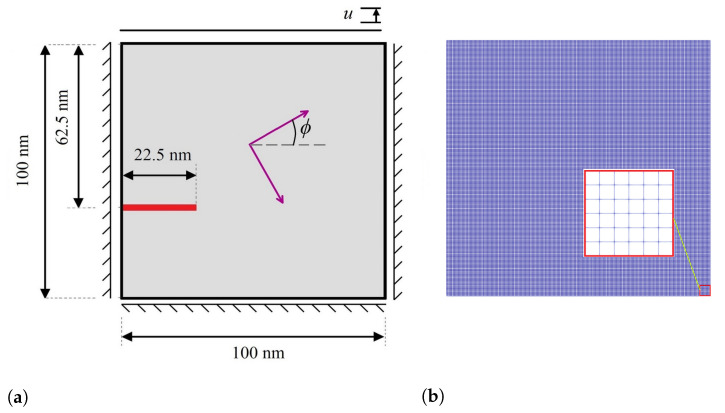
(**a**) Schematic representation of a specimen with initial crack under Mode I loading; (**b**) finite element mesh of the model.

In the present work, a square domain sized 100 nm × 100 nm discretized by 161,604 linear 4-node elements is considered, as shown in Figure 4a,b. The elasticity tensors are given by [55]

(22) $$\begin{array}{c} C_{11}^{A}=C_{22}^{A}=140\;\;\mathrm{GPa},\;\;\;\;\;\;\;\;\;\;\;\;C_{12}^{A}\;=C_{21}^{A}=84\;\mathrm{GPa},\;\;\;\;\;\;\;\;\;\;\;\;C_{33}^{A}=28\;\mathrm{GPa},\; \end{array}$$

(23) $$\begin{array}{c} C_{11}^{M}=C_{22}^{M}=154\;\;\mathrm{GPa},\;\;\;\;\;\;\;\;\;\;\;\;C_{12}^{M}\;=C_{21}^{M}=92.5\;\;\mathrm{GPa},\;\;\;\;\;\;\;\;\;\;\;\;C_{33}^{M}=31\;\mathrm{GPa} \end{array}$$

where *A* and *B* denote austenite and martensite phases, respectively.

In this work, in order to study the consequence of different crystal lattice orientation on the martensitic variants and crack behavior, a transfer operation of the tensorial quantities is demanded between the local coordinates of the crystal and the global coordinate system. The stress-free strain is transformed to the global coordinate system by means of the rotation operations given by

(24) $$\begin{array}{c} {\varepsilon_{i}}^{0}=R_{ik}R_{jl}{\varepsilon_{i}}^{00} \end{array}$$

where $R_{ik}$ denotes a rotation tensor, which for a grain having an orientation angle ϕ is defined by

(25) $$\begin{array}{c} R_{ij}=\left[\begin{array}{cc} \cos\phi & \sin\phi \\ -\sin\phi & \cos\phi \end{array}\right] \end{array}$$

and ${\varepsilon_{i}}^{00}$ is the eigenstrain tensor, which for the martensite variants with lattice orientation angle of 45° is given by

(26) $$\begin{array}{c} \varepsilon_{1}^{00}=\left[\begin{array}{c} -0.1\\ 0.1\\ 0 \end{array}\right],\;\;\;\;\;\;\;\;\;\;\;\;\;\;\;\;\;\;\;\;\;\;\;\;\;\;\;\;\varepsilon_{2}^{00}=\left[\begin{array}{c} 0.1\\ -0.1\\ 0 \end{array}\right] \end{array}$$

Furthermore, the calibration constants are selected as $\;k_{g}=0.6960$ and $k_{s}=1.3592$ with Gc=0.1JJm2m2 as a measure for the characteristic interface energy density [55]. The width of the transition zone is chosen as $L_{c}=1\;\mathrm{nm}$. Additionally, for the crack, the fracture energy Gs=1JJm2m2 and the crack width $L_{s}=1\;\mathrm{nm}$ are taken into account [60].

**Figure 5:**
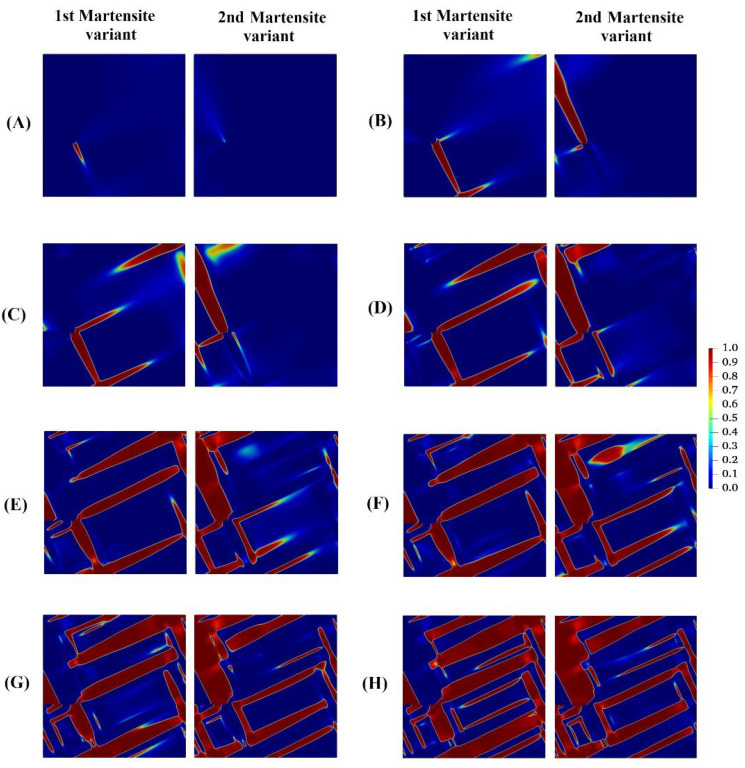
Evolution of two martensite variants prior to crack propagation in austenitic microstructure with crystal lattice orientation of 15${}^{0}$.

Figure 5 shows the evolution of two martensite variants prior to crack propagation in the austenitic microstructure with crystal lattice orientation of 15°. As can be observed from the figure, crack growth does not begin until MPT has grown almost completely through the microstructure. In order to interpret this phenomenon, note that prior to the propagation of the crack in the austenitic specimen, the external tension loading provides energy required for MPT. In other words, external loading as the main driving force is superior to the elastic energy and the gradient energy. On the other hand, MPT dissipates energy, which is, accordingly, not available for crack propagation. When the martensitic plates develop across the width of the microstructure, the elastic energy, which increases owing to the eigenstrain, can decrease on the macro-level where the microstructure is deformed. Then, this deformation of the microstructure induces extra stresses, in particular a shear loading, on the crack tip, which results in crack propagation.

**Figure 6:**
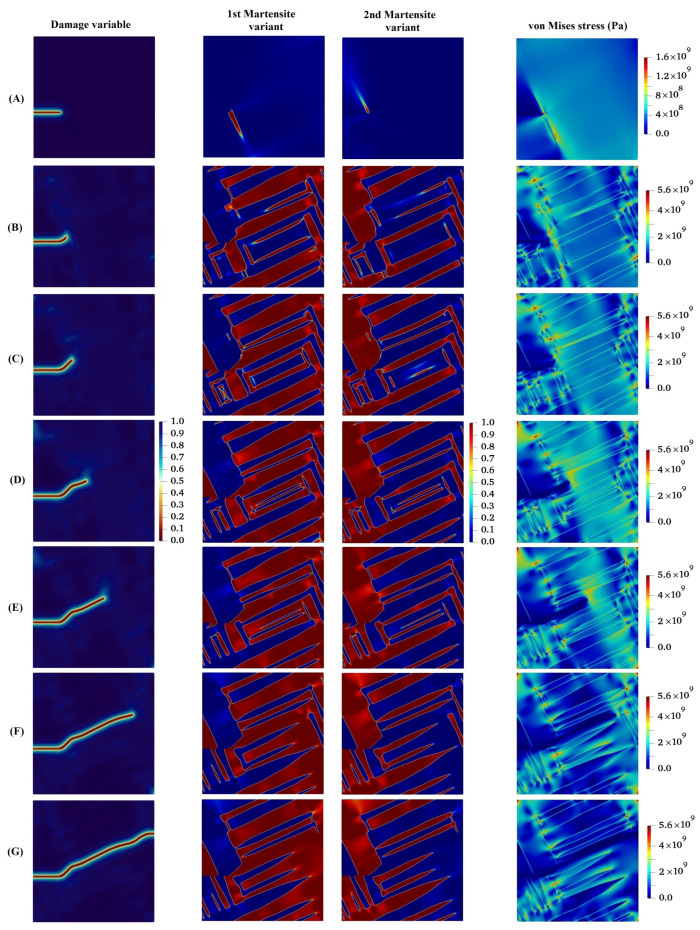
Evolution of damage variable, two martensite variants, and von Mises stress in the austenitic microstructure under Mode I loading with crystal lattice orientation of 15${}^{0}$.

Figure 6 demonstrates the growth of the initial crack and the evolution of martensite variants in the austenitic microstructure under Mode I loading with crystal lattice orientation of 15°. As can be seen in this figure, at the initial state by applying the loading, MPT occurs in the area adjacent to the crack tip owing to the high stress concentration at the initial crack tip. An analogous trend has been reported by Mamivand et al. [61] at the crack tip in tetragonal-to-monoclinic MPT. Subsequent to the initial formation of the martensite variants, the initial crack propagates in such a way that its path mainly depends on the feature of martensite variant formations, the orientation and direction upon which the martensite plates are aligned together, and the stress concentration between martensite plates. It can be concluded from Figure 6 that on the one hand, the stress concentration ahead of the crack tip affects the evolution of the martensite variants. On the other hand, the direction of crack growth alters with consideration of the formation of the plate-like martensites ahead of the crack tip. It is worth noting that the crack tends to propagate between martensite plates, which possess higher values of von Mises stress compared to other points.

**Figure 7:**
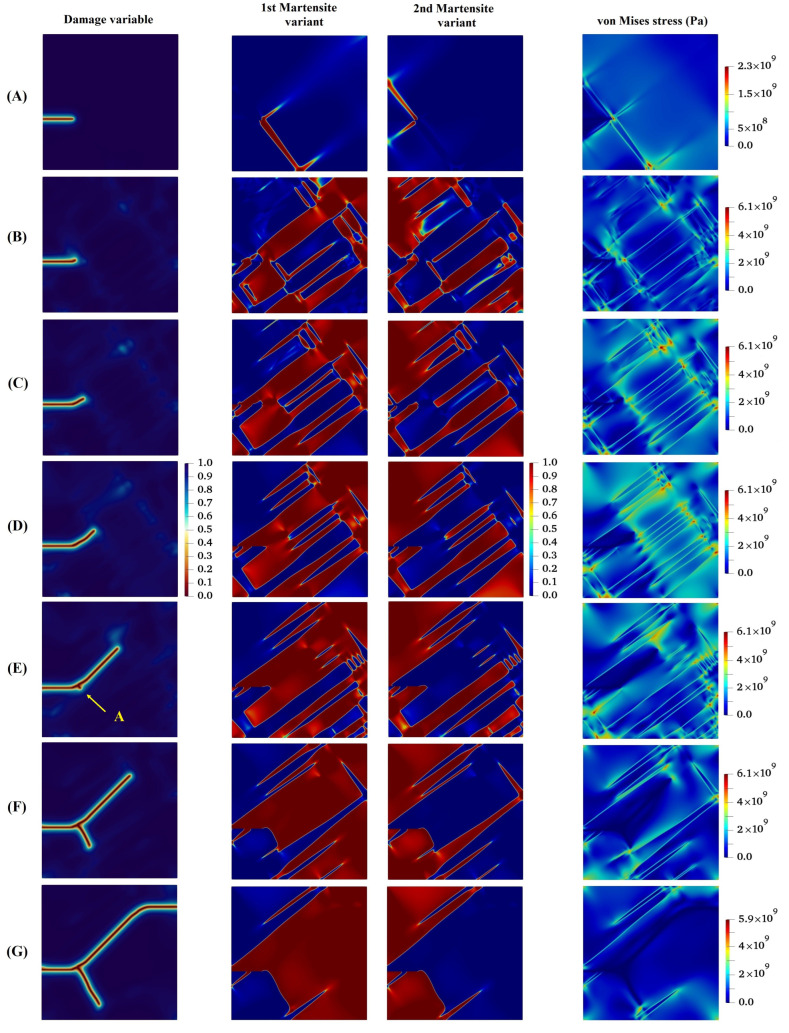
Evolution of damage variable, two martensite variants, and von Mises stress in the austenitic microstructure with crystal lattice orientation of 30${}^{0}$.

**Figure 8:**
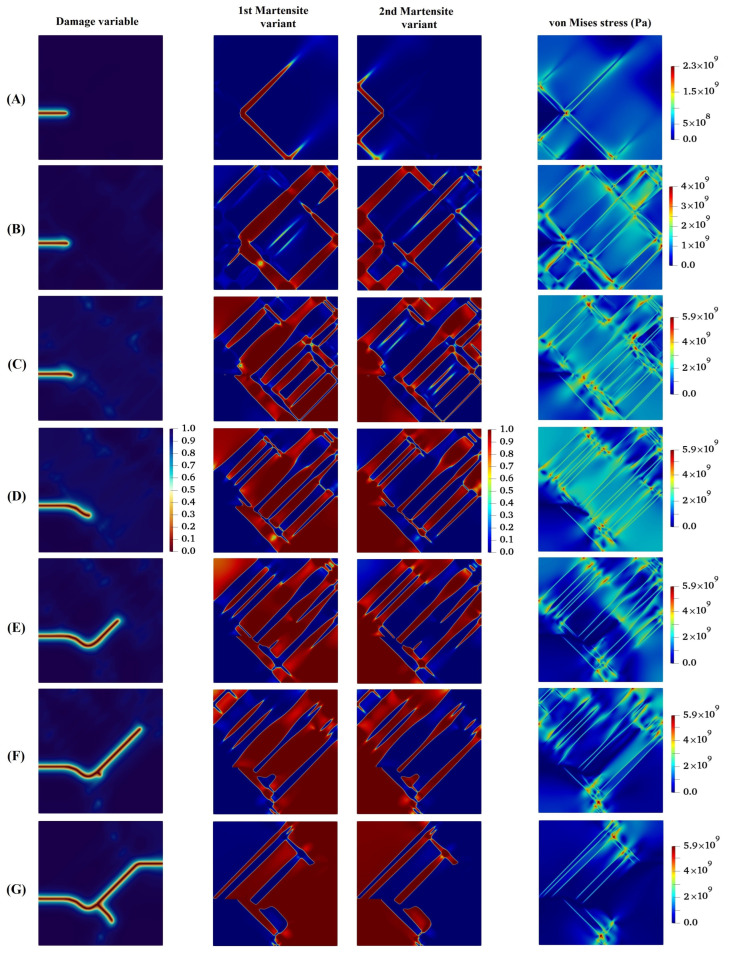
Evolution of damage variable, two martensite variants, and von Mises stress in the austenitic microstructure under Mode I loading with crystal lattice orientation of 45${}^{0}$.

**Figure 9:**
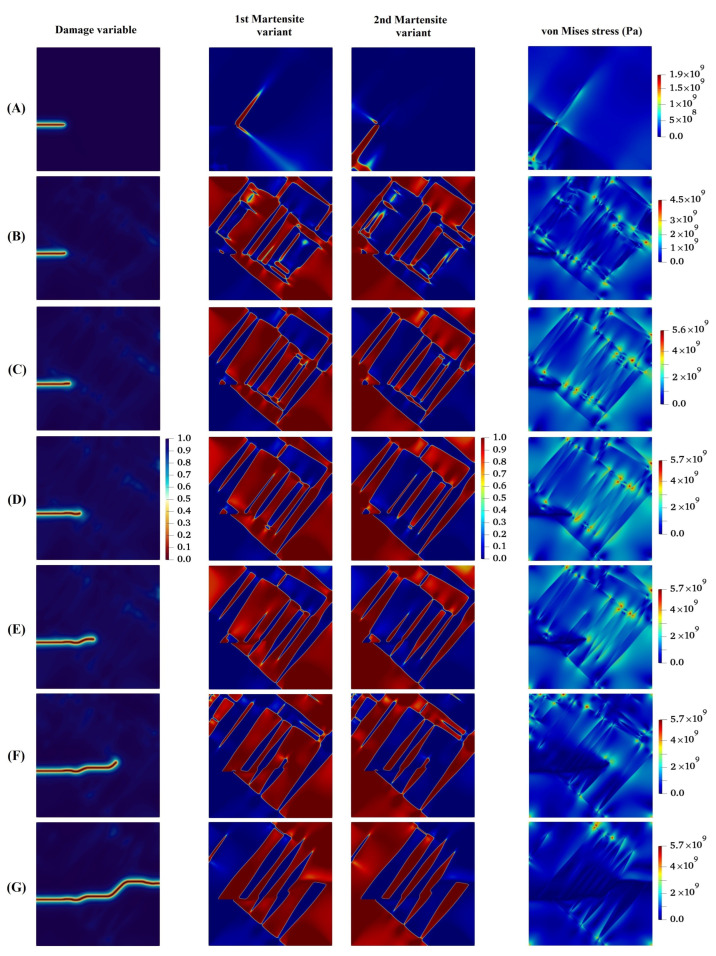
Evolution of damage variable, two martensite variants, and von Mises stress in the austenitic microstructure under Mode I loading with crystal lattice orientation of 60${}^{0}$.

**Figure 10:**
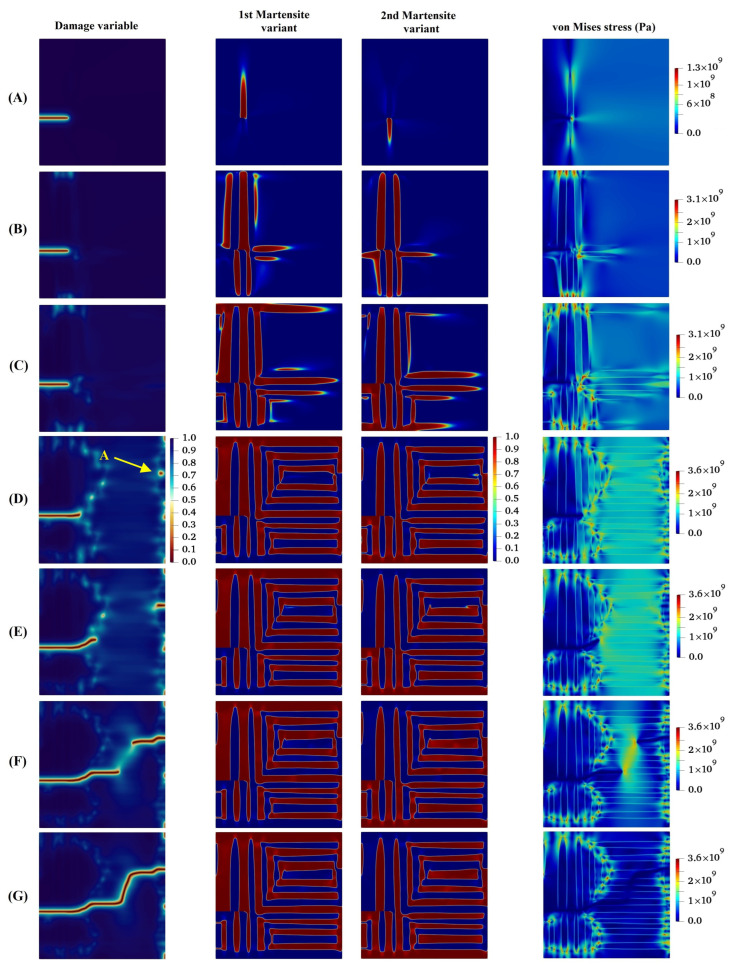
Evolution of damage variable, two martensite variants, and von Mises stress in the austenitic microstructure with crystal lattice orientation of 90${}^{0}$.

In the following, the crack propagation behavior and martensite variant evaluations for four different lattice orientation angles of 30°, 45°, 60°, and 90° are presented in Figure 7, Figure 8, Figure 9 and Figure 10, respectively, and compared with those with lattice orientation angle of 15°. It can be inferred from Figure 7 that as the crack propagates between layers of two martensite variants, as a result of high stress concentration at the intersection of martensite variants on the crack surface at Point A, crack-branching takes place. This observation can also be found in the crack propagation behavior of the microstructure with the lattice orientation angle of 45°, shown in Figure 8. Figure 9 shows that the crack for the case of the lattice orientation angle of 60° exhibits entirely different behavior compared with that of other lattice orientation angles, following a relatively straight path until the middle of the specimen. Another interesting point to mention is that the crack propagates with the martensite phase without reaching the boundary between the martensite variants, which coincides with the findings reported in the experimental work of Stolarz et al. [62].

It can be observed from Figure 10 that the crack initially grows within the martensite phase and deviates towards locations adjacent to the crack tip with high stress concentration. Afterwards, the crack tends to propagate between martensite variants, and at the same time, due to high stress concentration formed at the Point A, as shown in Figure 10, a new crack initiates and grows. After that, the two cracks approach each other irrespective of the boundary between martensite variants and eventually intersect.

**Figure 11:**
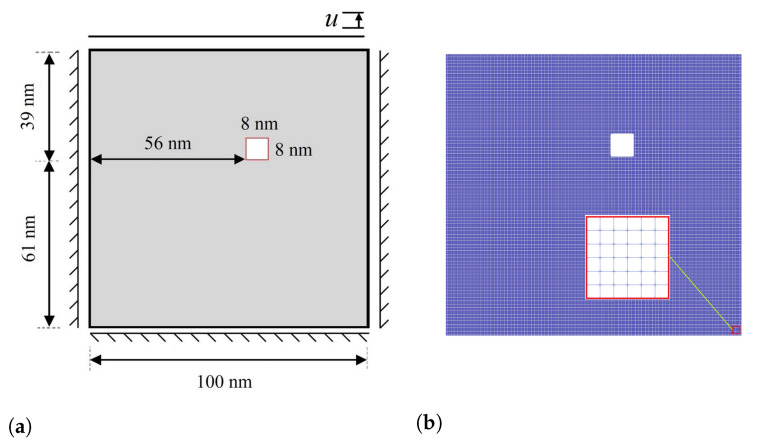
(**a**) Configuration of the specimen with a geometric inhomogeneity under Mode I; (**b**) finite element mesh of the model with an inhomogeneity.

**Figure 12:**
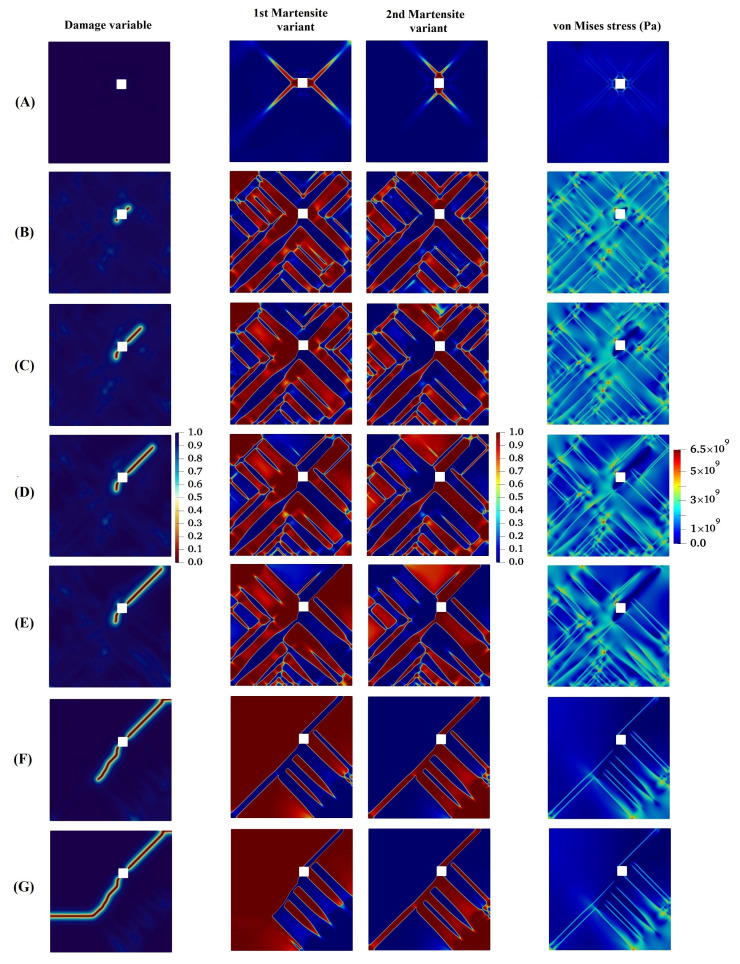
Evolution of damage variable, two martensite variants, and von Mises stress in the austenitic microstructure with a geometric inhomogeneity.

**Figure 13:**
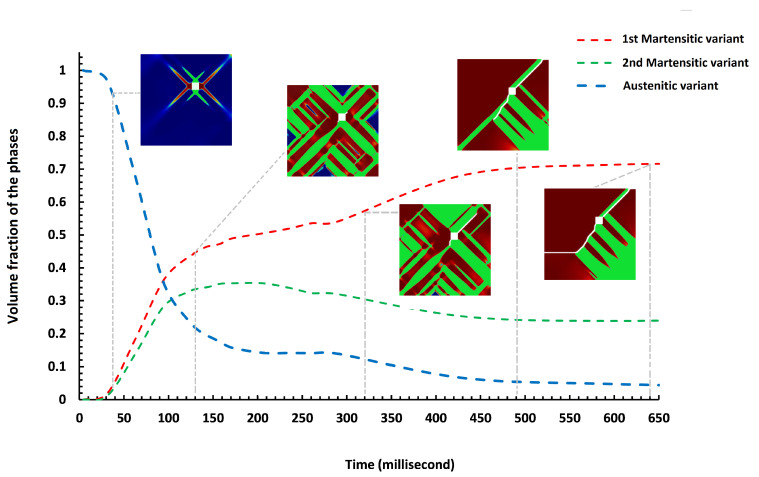
Variation of volume fraction of austenite and martensite phases for the austenitic specimen with a geometric inhomogeneity.

In the next example, we consider an imperfection in the specimen, such as porosity, which is the main location for the stress concentration, leading the crack to onset and propagate thorough the material until eventual failure. In this case, as a result of the high stress concentration, a significant stress field is formed, which supplies the energy required for MPT during a stress-induced process. In order to better investigate the concept, a square geometric inhomogeneity is created in the specimen, as shown in Figure 11a. Evolution of the damage variable, two martensite variants, and von Mises stress in the austenitic microstructure with a geometric inhomogeneity is shown in Figure 12. As can be seen, there is no initial crack, as opposed to previous examples. However, the numerical technique presented is capable of modeling the initiation of the crack. The initial martensitic plates are formed as a consequence of MPT in the material, and cracks start appearing in the corners of the square and propagate outward from there. Figure 13 graphically demonstrates the formation of martensite variants in the austenitic field. The first variant is the dominating variable in this structure, as shown in the diagram, occupying a larger percentage of volume at all times. Up to 150 milliseconds, both martensite variants have an ascending trend, with the first and second variants of martensite having volume fractions of 48 and 35 percent, respectively.

**Figure 14:**
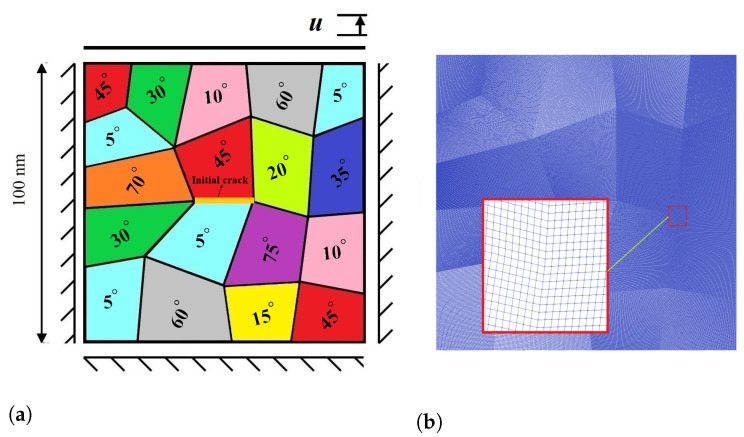
(**a**) Configuration of the polycrystalline specimen; (**b**) finite element mesh of the polycrystalline model.

**Figure 15:**
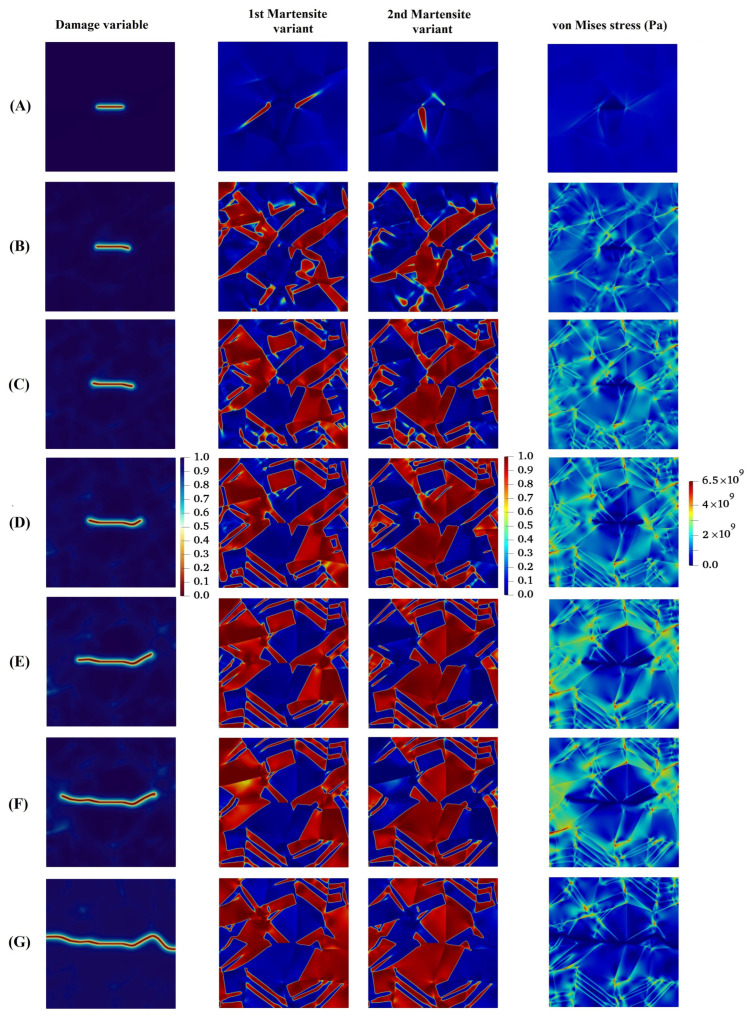
Evolution of damage variable, two martensite variants, and von Mises stress in the polycrystalline microstructure.

In this part, we turn our attention to coupled problem of MPT and fracture in polycrystalline microstructures. To this end, a polycrystalline model is built. This microstructure is arranged completely randomly so that in each crystal there is the potential for the growth of martensitic layers at different angles, as can be seen in Figure 14a. In this investigation, the effects of the grain boundaries are not been considered as separated behaviors. Figure 15 shows that the formation of martensitic layers begins under a stress-induced process from the crack tip. Each crystal provides a condition for the growth of martensitic layers at different angles, which are formed prior to the growth of cracks in the initial martensitic structure. After the formation of the initial martensitic structure in the material, the crack begins to propagate through the material. It is worthwhile to note that crack growth mainly depends on several factors, such as individual crystal orientation, the arrangement of the martensitic layers, maximum stresses, and the stress concentration in the regions.

## 5. Conclusions

In this work, a coupled problem of crack nucleation and propagation and two-variant MPT was investigated based on PF approach-based FE formulations. The model established includes a coupled system of three TDGL questions that describe the evolution of the damage variable and two martensite variants in the quasi-static equilibrium equation. This work has accounted for the positive dilatational component of the transformation strain, which accompanies the MPT from austenite to martensite phase and leads to an eigenstrain within the martensitic phase. Since the eigenstrain results in both tensile and compressive loads, the model considers the sign of the dilatational component. In particular, this study concentrated on the interactions between microcrack initiation and propagation and 2D phase transformation. The main results can be summarized as follows:

- The results reveal that crack growth does not begin until MPT has grown almost completely through the microstructure. This can be mainly attributed to the fact that MPT dissipates energy, making the energy unavailable for crack propagation.
- Subsequent to the initial formation of the martensite variants, the initial crack propagates in such a way that its path mainly depends on the feature of martensite variant formations, the orientation and direction upon which the martensite plates are aligned, and the stress concentration between martensite plates.
- The results showed that for lattice orientation angles of 30${}^{0}$ and 45${}^{0}$, as the crack propagates between layers of two martensite variants, due to high stress concentration at the intersection of martensite variants on the crack surface, crack branching takes place.
- For the lattice orientation angle of 90${}^{0}$, it can be concluded that the crack tends to propagate between martensite variants, and at the same time, due to the high stress concentration formed at a location far from the main crack, a new crack initiates and grows. After that, the two cracks approach each other irrespective of the boundary between the martensite variants and eventually intersect.
- The last example demonstrates one of the significant advantages of the phase-field method in comparison to other methodologies. This method, in contrast to the majority of other techniques, has the ability to identify places in the material with the potential to initiate cracks. The model illustrates that martensitic phase change can start even in the absence of martensitic nuclei when subjected to stress concentrations due to geometric heterogeneity. Then, the phase change can be extended to the entire component. The fracture also commences nucleation and propagates through the material, and this process continues under a fully coupled martensitic transformation to final failure.

## Data Availability

The data presented in this study is available from the corresponding authors upon reasonable request.
